# Reducing meat consumption through default nudging: a field study

**DOI:** 10.3389/fpsyg.2025.1439641

**Published:** 2025-04-30

**Authors:** Tamar F. Ardesch, Sandra Klaperski-van der Wal, Sari R. R. Nijssen, Barbara C. N. Müller

**Affiliations:** ^1^Behavioural Science Institute, Radboud University Nijmegen, Nijmegen, Netherlands; ^2^Department of Emotion, Cognition, and Methods in Psychology, Urban and Environmental Psychology Group, Faculty of Psychology, University of Vienna, Vienna, Austria

**Keywords:** default nudging, intervention research, meat consumption, environmental impact, behaviour change

## Abstract

Meat consumption negatively impacts ecological sustainability, health, and animal welfare. Research suggested promising effects of re-designing product arrangements so that vegetarian items become the default. However, whether default nudging leads to actual behaviour change in the context of meat consumption, and whether these effects are sustainable on the long-term remains unknown. Therefore, this field experiment investigated (a) the effect of vegetarian default nudging on food choices in a real-life setting, and (b) potential long-term associations between vegetarian defaults, food choices, and psychological resistance (i.e., reactance and inertia). A vegetarian default intervention was applied in a university cafeteria. Behavioural data (cafeteria sales data regarding meat and vegetarian purchases; *N* = 4,099) was collected before (T0; baseline), right after implementation (T1), and 10 weeks after implementation of the intervention (T2). Additionally, survey data was collected at T1 and T2 to assess potential psychological side-effects, such as resistance to the intervention. Results indicate that vegetarian default nudging was highly effective at changing food choices, with more than twice the number of vegetarian items sold relative to baseline. Moreover, in the default nudging condition, the number of meat items decreased to less than a third of the baseline measurement. At the same time, the survey data revealed no psychological side-effect of the intervention on reactance or inertia. This was stable over time. Our research offers empirical support for the effectiveness of a non-coercive strategy to change consumer behaviour towards more sustainable, animal friendly, and healthier food choices.

## Introduction

### Reducing meat consumption through default nudging: a field study

Global meat consumption nearly doubled in the last 20 years (Parlasca and Qaim, 2022). This increase comes at the cost of human health (e.g., zoonosis, cancers, cardiovascular diseases and antibiotic-resistant pathogens; Almashhadany, 2021; Godfray et al., 2018; González et al., 2020; Monger et al., 2021), the environment (i.e., significant resource demands and emissions; González et al., 2020; Lehner et al., 2016; Machovina et al., 2015) and animal welfare (i.e., unhygienic and constrained living conditions; Appleby et al., 2004; Fiber-Ostrow and Lovell, 2016; and the abuse and slaughter of animals; Eisnitz, 2009; Roser, 2023). Although many individuals are interested in reducing their meat consumption, accomplishing a complete absence of eating meat appears to be difficult: while 31% of the population in the European Union indicate to be willing to reduce their meat intake, 13% indicate to avoid meat completely (Eurobarometer, 2023; EPRS, 2020). Importantly, default nudging is a well-established behavioural intervention strategy particularly relevant for deeply entrenched habits—such as meat consumption (Meier et al., 2022). Therefore, the goal of the current study was to investigate whether a default nudging intervention could be effective at changing food choices. Specifically, we tested this in a field setting, with measurements of real behaviour (i.e., sales data) and surveys to investigate how behavioural choices relate to potential psychological side-effects such as resistance. Moreover, to assess the potential long-term effects of default vegetarian nudging, data was collected at three time points across a period of 4 months.

### The intention-behaviour gap and default nudging

The intention-behaviour gap has been observed in a wide variety of health-related and sustainable behaviours (Blake, 1999; Carrington et al., 2014; Fishbein and Ajzen, 1977; Knowles and Linn, 2004), such as exercise (Rhodes and de Bruijn, 2013), organ donation (Crawshaw et al., 2022), electronic waste recycling (Echegaray and Hansstein, 2017), and sustainable fashion (Park and Lin, 2020). Of particular relevance to the current study, research has also identified an intention-behaviour gap specifically in the context of meat consumption. For example, research by Arnaudova et al. (2022) as well as Laffan et al. (2023) shows that, respectively, half and one-third of participants in their studies failed to align their intention to reduce their meat intake with actual dietary choices, with taste and cooking habits being ultimately more important determinants for eating behaviour (Laffan et al., 2023).

A specific behavioural intervention which can be used to reduce meat consumption among individuals who encounter difficulties aligning their intentions and behaviour, is vegetarian default nudging (Campbell-Arvai et al., 2014; De Vaan et al., 2019). A systematic review of twelve articles indeed suggests that default nudging is an encouraging intervention method to decrease meat consumption (Meier et al., 2022). This default nudging is a cost-effective and non-disruptive strategy that changes behaviour through subtle environmental adjustments to increase the chance for the desired behaviour, while preserving individuals’ freedom of choice (Campbell-Arvai et al., 2014; Hertwig and Grüne-Yanoff, 2017; Kollmuss and Agyeman, 2002; Papies, 2017; Thaler and Sunstein, 2008). A default option is the standard option provided when individuals do not explicitly request alternatives (Thaler and Sunstein, 2008). Other options (e.g., meat items) are thus available, but they require proactive action from the individual. This makes vegetarian defaults an effective strategy for attempting to change the behaviour of individuals who are opposed to changing their food habits as well. As humans generally prefer convenience (Farquhar and Rowley, 2009), it could be that opting for the behaviour requiring the least effort (i.e., choosing the vegetarian default option) outweighs the desire for an initially intended but more demanding option (i.e., proactively inquiring about a meat or fish item) even in individuals who are initially opposed to such changes. This was demonstrated in a study by Hielkema et al. (2022), in which the vegetarian default enhanced the choice for the vegetarian option more strongly among individuals with no intention to reduce their meat intake than among those who already aimed at reducing their meat consumption. Additionally, since the desired behaviour is not forced, there is less chance of non-compliance (Knowles and Linn, 2004) or backlash effects (i.e., increasing meat consumption as a reaction to feared or possible meat restrictions; Lombardini and Lankoski, 2013). Thus, freedom of choice is preserved using vegetarian defaults, whilst the chances of seeking the less desired alternative option decrease (Hertwig and Grüne-Yanoff, 2017).

Whilst transitioning to entirely plant-based defaults would offer the greatest benefits in terms of ecological sustainability, health, and animal welfare (Fehér et al., 2020; Willett et al., 2019), it may be considered too radical by the population consuming animal-derived products, as vegan or plant-based diets are often even more controversial than vegetarian diets (e.g., De Groeve and Rosenfeld, 2022; Gregson et al., 2022; MacInnis and Hodson, 2017). As previous research has shown (e.g., Betz et al., 2022; Campbell-Arvai et al., 2014; De Vaan et al., 2019; Gravert and Kurz, 2021; Meier et al., 2022; Taufik et al., 2022), presenting a vegetarian option as the default can increase both the selection of meat-free dishes and the demand for vegetarian options when people eat out of home. Therefore, changing the default food items into vegetarian food items, seems currently a more realistic approach for increasing the demand for vegetarian dishes and reducing the demand for meat and fish dishes, without demanding a too substantial or controversial shift.

### The current study

Recent studies investigating vegetarian defaults in either US or European contexts were conducted solely in online and laboratory settings and were not longitudinal (e.g., De Vaan et al., 2019). Furthermore, studies that used a more naturalistic set-up kept certain unnaturalistic constraints in their design. For instance, the study by Campbell-Arvai et al. (2014) was conducted in dining halls of universities, but the set-up lacked the real-life consequences of their choice, that is, paying and receiving the food. In contrast, the field study lasting for four weeks by Taufik et al. (2022) did include the real-life consequences but used the vegetarian default solely on the week menu, leaving the regular menu unchanged, with meat and fish options available. Furthermore, the field study by Gravert and Kurz (2021) solely examined a menu with two options available (i.e., meat and fish vs. vegetarian and fish), with the footnote that, on request, a vegetarian or meat dish could be prepared. In sum, the existing evidence is limited with regard to the internal and external validity, as well as the generalizability of their results. Methodological improvements, such as longitudinal data collection and a more realistic range of menu options, are needed to draw effective conclusions about the impact of vegetarian default nudging on food choice.

We address the shortcomings and add to earlier studies, as our study tests a default nudge in a field setting, with measurements of real behaviour (i.e., sales data) and survey data to investigate how behavioural choices relate to potential psychological side-effects such as resistance. Moreover, to assess the potential long-term effects of default vegetarian nudging, data was collected at three time points across a period of 4 months. We, thus, aim to increase internal and external validity, as well as the generalizability, which might contribute to clarify how vegetarian defaults can be implemented as a subtle, non-disruptive, and cost-efficient, yet effective and feasible behaviour change strategy.

The current study set out to investigate the role of vegetarian defaults on meat consumption on the short- and long-term. We conducted a longitudinal field study in a university cafeteria over a period of 16 weeks, assessing actual behavioural choices. At three timepoints, sales data for vegetarian and non-vegetarian items (i.e., behavioural data) were collected to monitor behavioural changes. With regard to the behavioural data and building upon prior research (Campbell-Arvai et al., 2014; De Vaan et al., 2019; Gravert and Kurz, 2021; Hielkema et al., 2022; Meier et al., 2022; Taufik et al., 2022), we hypothesized that changing the default items into solely vegetarian items would be associated with an increase in the sales of vegetarian items, alongside a decline in the sales of fish and meat items (H1a). Furthermore, we expected this pattern to remain stable over time at the follow-up measure (H1b).

In addition to these central hypotheses, we were also interested in exploring two potential psychological side-effects of a default nudging intervention. First, an often-noted potential negative side-effect of default nudging is reactance – which seems pertinent particularly in the domain of food choices and was therefore included in the survey (e.g., Rosenberg and Siegel, 2018; Van Dinther, 2015). Second, a potential positive side-effect might occur for customers who experience inertia regarding their meat consumption. For them, our default intervention might act as nudge towards a behavioural choice that they consciously support already, but find difficult to achieve (Joyner-Grantham et al., 2009; Knowles and Linn, 2004; Polites and Karahanna, 2012). All hypotheses and research questions were preregistered on the Open Science Framework.^1^

## Method

This study was conducted at the central cafeteria of the Faculty of Social Sciences ‘De Iris.’ Ethical approval from the Ethics Committee of Radboud University Nijmegen was obtained before data collection: ECS-LT-2022-10-12-11646. The analyses and materials are available on the Open Science Framework (see text footnote 1).

### Participants

#### Behavioural data

For the behavioural data, the cafeteria provided count data on the items sold during lunch breaks, specifying whether items were non-vegetarian (meat and/or fish) or vegetarian (including vegan) items. More precisely, the behavioural data consisted of three lists detailing the total quantities sold of each item over the periods of 2 weeks. Meat/fish items had approximately the same price as vegetarian/vegan items. Snacks (e.g., candy bars and fruits) and breakfast items (e.g., yoghurt or croissants) were not included in the list. Furthermore, due to practical constraints, it was not feasible to obtain a list with all the separate transactions, specifying the number and sort of items sold within each purchase. Thus, it is important to note that it is unclear whether one customer purchased solely one or multiple items. Whilst this data collection process involved human participants, no personal identifying information was gathered, hence informed consent was not obtained for assessing behavioural data. Each datapoint represents one food item that was sold in the between 11:45 and 13:30 during one of the three data collection periods. In total, there were 4,099 items sold, with 1,245 items sold at T0, 1493 items at T1, and 1,361 items at T2. A post-hoc sensitivity analysis revealed that, at alpha = 0.05, power = 0.95, and the degrees of freedom set to 2, the minimum test statistic detectable by this research design and sample size would be $\chi^{2}$ = 5.99.

#### Survey data

The initial sample that participated in the survey consisted of *N* = 314 customers. *N* = 87 participants (27.71%) had incomplete data on the relevant variables due to a technical error (i.e., the items assessing reactance, inertia, and covariates) and were therefore excluded from further analysis. This resulted in a final sample of *N* = 227 participants (191 women, 29 men, 3 non-binary, and 4 of unknown gender) between 18 and 37 years old (*M*age = 20.71, *SD*age = 2.58). The sample size was determined based on an effect size of $f^{2}=0$ .15 (medium effect size; Campbell-Arvai et al., 2014), requiring a sample size of at least 66 participants per time point (i.e., a total of at least 198 participants) to achieve a statistical power of 95%.

Participants were recruited in the cafeteria. Eligible participants had to be regular customers of the cafeteria (i.e., purchase meals at least once a month). Participants were permitted to participate only once per timepoint to ensure statistical independence of the data points. Participants were self-selected and received a snack (i.e., a chocolate bar or candy) for their participation. The completion of the survey lasted approximately 5 min. Furthermore, participants could submit their email address to participate in a lottery to win a 50€ voucher.

### Procedure

**Figure 1:**
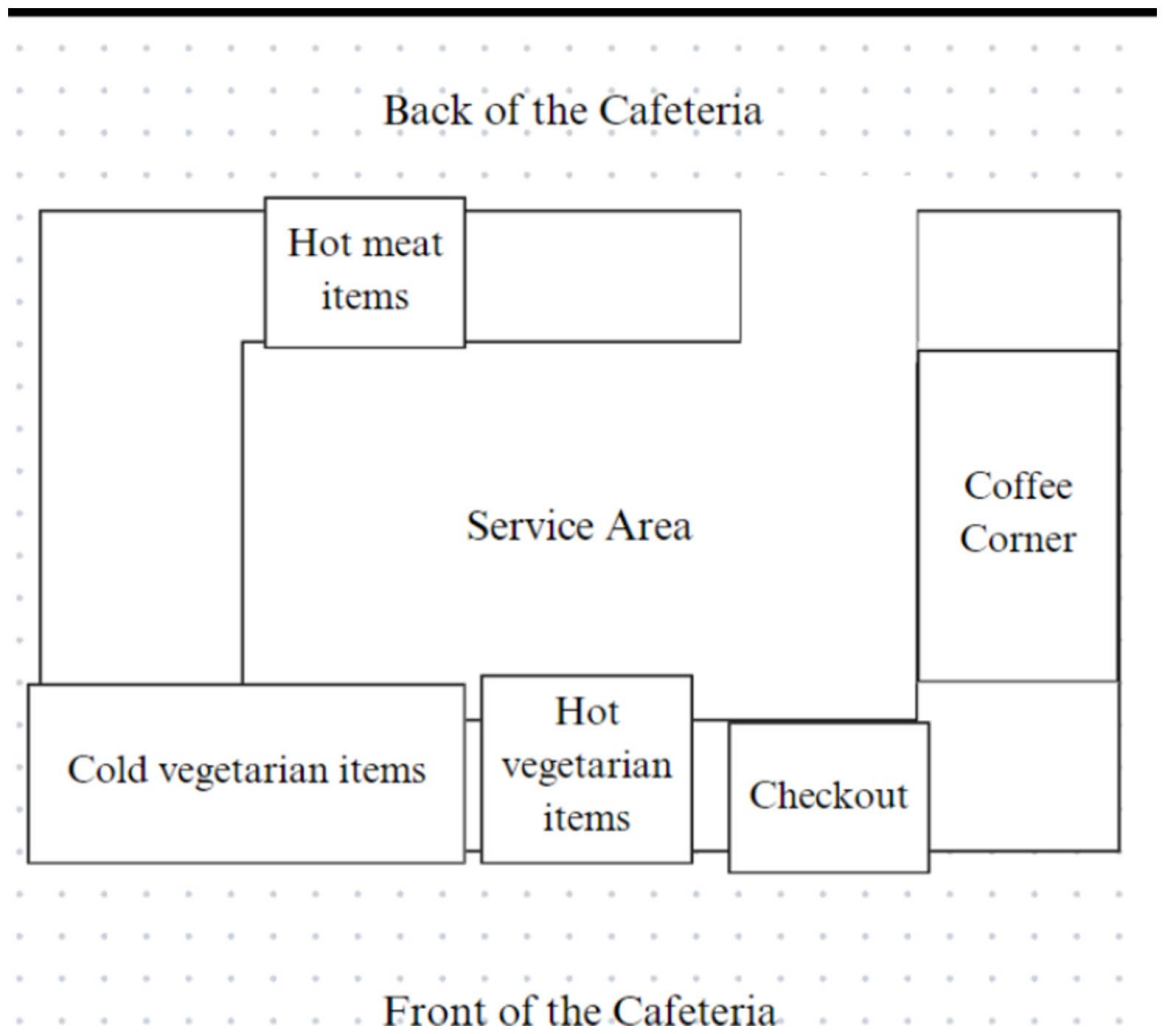
Map of the cafeteria. Customers approached items from the front area of the cafeteria. Only staff was allowed to access the service area.

**Figure 2:**
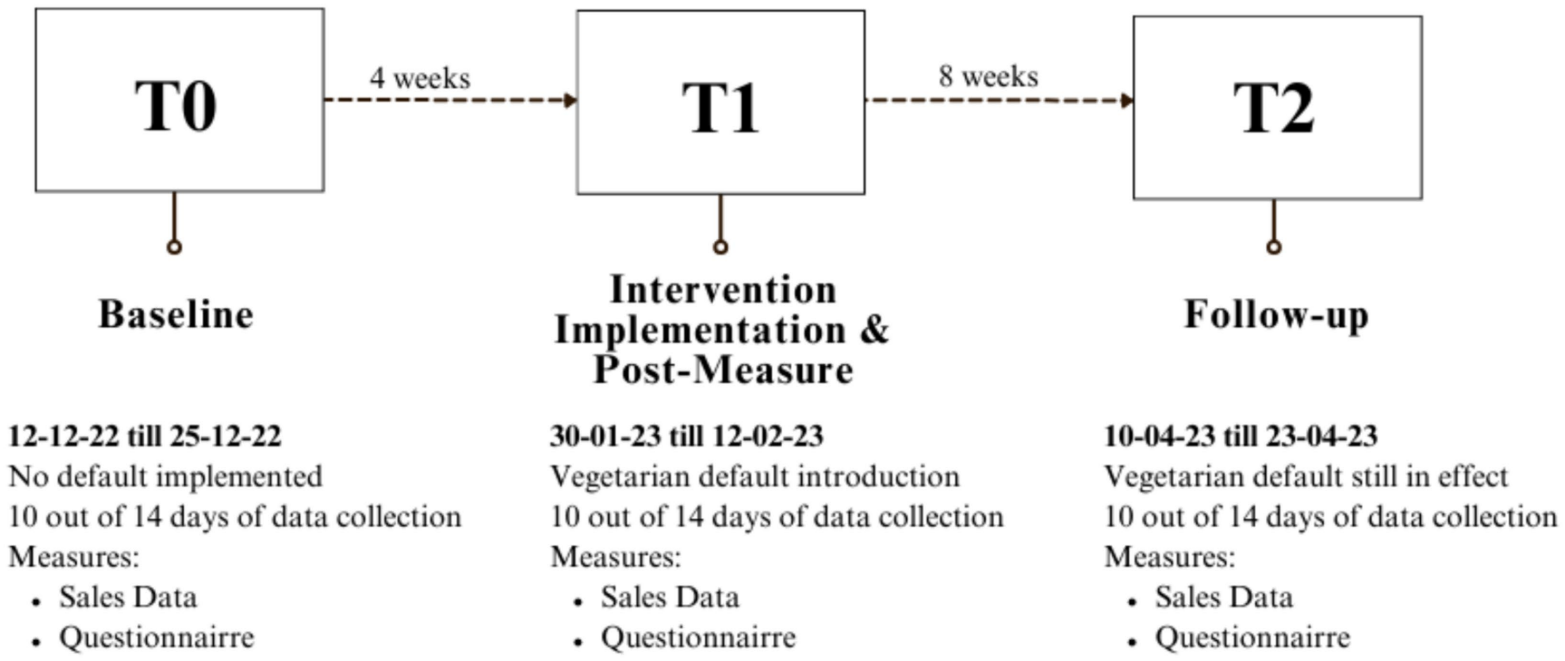
Timeline visualising the measures and intervention. The experiment lasted 16 weeks from start to finish, with 2 weeks per time point for data collection.

This field study applied a default nudge intervention. To create the vegetarian default, the displayed food items in the cafeteria’s showcase (i.e., the defaults) were changed to exclusively feature vegetarian items. Fish and meat items remained available but were listed on screens above the counter and had to be specifically requested; they were no longer displayed in the showcase (see OSF for the list of the available food items per timepoint). Due to practical constraints, warm snacks containing meat were placed in the back of the cafeteria instead of under the counter (see Figure 1). Thus, these were not completely out of sight, but the warm vegetarian snacks were more visible as they were displayed in front of the customers on top of the front counter. Data were collected during lunch breaks from 11:45 to 13:30 for 2 weeks per timepoint. Data collection occurred at three different timepoints (see Figure 2): the baseline measure, 6 weeks before the intervention (T0); the post-measure, immediately after the start of the intervention (T1); and the follow-up measure, 10 weeks after the default intervention was introduced (T2). These periods were selected while considering holidays and exam weeks to ensure an accurate representation of the entire year in terms of visitors and sales.

In addition to the behavioural data, visitors of the cafeteria were invited to participate in a survey. If eligible, they scanned a QR-code presented by the researcher and were directed to the online survey (Qualtrics, 2023), gave informed consent, and were informed about the study goal. After survey completion, participants were thanked, debriefed, and rewarded. Due to practical constraints, it was not feasible to directly invite customers of the cafeteria after purchase, as this would have disrupted the workflow of the cafeteria. Thus, it is important to note that the sales data and the survey data are not connected, that is, the participants who filled out the survey were not necessarily the ones purchasing food items during the measurement period.

## Materials

### Survey

*Dietary Preferences.* The survey^2^ was phrased in English and contained a semi-closed-ended question on whether respondents followed a certain diet (Kloosterman et al., 2021) to check if the distribution of the diets were equal across the three different measures.

*Reactance.* Reactance was assessed with two scales: perceived threat of freedom and anger (Dillard and Shen, 2005). The items were adapted from the original scales in two significant ways. First, we used the phrase “the food options visible in the counter” for each item (e.g., “The food options visible in the counter threatened my freedom of choice”). Second, we phrased each item twice: once to assess the general perceived threat to freedom and anger regarding the selection of food options, and once to assess the perceived threat to freedom and anger as regarding the board of the cafeteria and its decision-making regarding the food options (e.g., “By offering certain food options, the board of the cafeteria threatened my freedom of choice”). These modifications resulted in a total of eight items per scale, resulting in a total of 16 items measuring reactance. Participants could respond on a 5-point Likert scale (1 = totally disagree, 5 = totally agree). Reliability measures indicated acceptable reliability (Cronbach’s *α = 0*.73) for the threat of freedom scale and an excellent reliability for the anger scale (Cronbach’s *α = 0*.90) related to questions about food options. Meanwhile, questions related to the cafeteria board showed good reliability for the threat of freedom scale (Cronbach’s *α* = 0.81) and excellent reliability for the anger scale (Cronbach’s *α* = 0.92).

*Inertia.* Inertia was assessed using six items (Cronbach’s *α* = 0.90), based on Han et al. (2011). For example: “I think increasing the number of vegetarian meals I consume would be better, but I fail to do so.” Participants responded on a 5-point Likert scale (1 = totally disagree, 5 = totally agree).

*Demographic Information & Covariates.* Participants provided their age numerically and indicated their gender with answer options “Male,” “Female,” “Other ….,” and “Prefer not to say.” In addition, eight other potential covariates were measured and analysed. Since these analyses were of a more exploratory nature, and none provided significant or otherwise relevant insight into the psychological dimensions of our study, we decided to omit them from the main text of this manuscript for the sake of conciseness. However, we included a full overview of the covariates and the relevant analyses on the Open Science Framework.

### Data analysis

The data were analysed using the statistical software R Core Team (2023) and JASP Team (2023).

#### Principal component analyses

Initially, several Principal Component Analyses were conducted with JASP Team (2023) to examine item relationships. The first analysis explored the relation between the eight items of the reactance anger scale and the eight items of the threat of freedom scale. The results indicated that reactance consisted of these two components (i.e., the perceived threat of freedom and anger scales) separately. However, as questions for the anger and threat of freedom scales were posed for both food options and the cafeteria board, we decided, based on the definition, to analyse them separately. This decision resulted in reactance comprising four subscales (i.e., two for threat of freedom and two for anger). Additionally, the analysis revealed that the seven items constituting the covariate for reactance attitude loaded onto one component. Moreover, the six items of inertia loaded onto one factor as well, separately for each scale.

#### Data pre-processing and assumption checks

To prepare the data for processing, first, the lists containing the total quantities sold of each item were used to compute two sum scores per timepoint, meaning one sum score for the total of vegetarian items sold (including vegan items), and one sum score for the total of sold items containing meat. It is important to note that no items containing fish were sold over this period. Similarly, sum scores of the answers were computed per timepoint for the dependent variables of the four reactance subscales and the inertia variable.

Subsequently, assumption checks for the chi-square tests were investigated. Violations are noted when relevant. Two outliers were detected in the reactance and inertia scales, respectively. However, since these outliers did not contain values outside of the possible score range, we decided to include the data of these participants in the analyses. Finally, the results of a one-way ANOVA and two Fisher’s Exact Tests revealed that the three samples of the survey did not significantly differ regarding age, *F*(2, 224) = 1.296, *p* = 0.276, gender (*p* = 0.370), and diet (*p* = 0.362).

#### Main analyses

*Behavioural Data.* Initially, three chi-square tests of independence were employed to examine the relation between behavioural data (i.e., the number of units sold of vegetarian and meat items) and intervention implementation (indicated by the variable of time). Specifically, the first chi-square test compared T0 with T1 regarding the distributions of the number of purchased vegetarian and meat items, the second test examined the relationship between T0 and T2 in the same manner, and the third test investigated whether the distributions of the vegetarian and meat items differed for T1 and T2.

### Survey data

*Reactance.* To investigate whether reactance changed over time after the intervention implementation, a Multivariate Analysis of Variance (MANOVA) was conducted in which the perceived threat of freedom (regarding food options and cafeteria board) and feelings of anger (regarding food options and cafeteria board) were set as outcome variables, whilst time (T0, T1, and T2) was used as a categorical predictor.

*Inertia.* To explore the relation between the intervention and inertia over time, a one-way Analysis of Variance (ANOVA) was conducted. Inertia was set as outcome variable, whilst time (T0, T1, and T2) was used as a categorical predictor.

## Results

### Main analyses

#### Behavioural data

**Figure 3:**
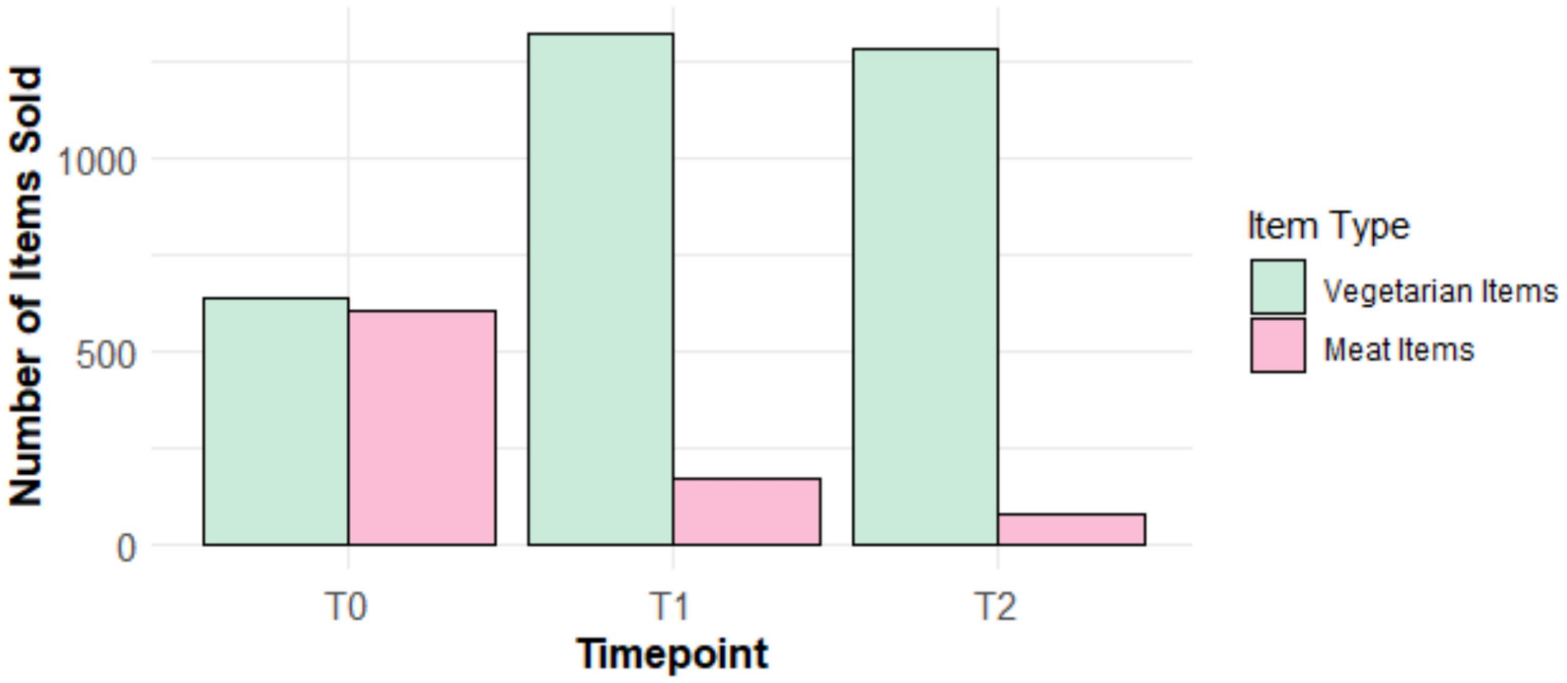
Visualisation of the sales per item type-over-time. To baseline measure, T1 = post-measure, T2 = follow-up measure.

Results are summarized in Figure 3. The first chi-square test of independence demonstrated that the distribution of the number of sold vegetarian and meat items differed between T0 and T1, $\chi^{2}$ (1, *N* = 2,738) = 465.57, *p* < 0.001, Cramér’s *V = 0*.41, which is considered a strong effect. At the post-measure implementation, the sales of the number of vegetarian items increased (*n*_T0vegetarian_ = 641 versus *n*_T1vegetarian_ = 1,325), while the sales of the number of meat items decreased (*n*_T0meat_ = 604 versus *n*_T1meat_ = 168) as compared to the baseline measure.

The second chi-square test of independence yielded a significant difference between T0 and T2 in the pattern of the number of vegetarian and meat items sold, $\chi^{2}$ (1, *N* = 2,606) = 615.96, *p* < 0.001, Cramér’s *V = 0*.49, which is considered a strong effect. To be more precise, the number of vegetarian items sold was higher in T2 as compared to T0 (*n*_T0vegetarian_ = 641 versus *n*_T2vegetarian_ = 1,283), whilst the number of meat items exhibited an opposite trend (*n*_T0meat_ = 604 versus *n*_T2meat_ = 78).

The final chi-square test of independence elucidated that T1 and T2 slightly differed regarding the sales of the vegetarian and meat items, $\chi^{2}$ (1, *N* = 2,854) = 27.56, *p* < 0.001, Cramér’s *V = 0*.10, which is considered as a small effect. This analysis revealed that there was a small decrease from T1 to T2 in the sales of vegetarian items (*n*T1vegetarian = 1,325 versus *n*T2vegetarian = 1,283) and a slightly stronger decrease in the sales of meat items (*n*_T1meat_ = 168 versus *n*_T2meat_ = 78).

#### Survey data

An overview of all descriptive statistics of the study is depicted in Table 1. All variables demonstrated deviations from normality. However, since the MANOVA and ANOVA are robust analyses which are not very sensitive to these deviations (Glass et al., 1972), we assumed little implications for our results and continued analyses as pre-registered.

**Table 1:** Descriptive statistics of the dependent variables and covariates.

Dependent variable	Overall		T0		T1		T2	
	*M*	*SD*	*M*	*SD*	*M*	*SD*	*M*	*SD*
Perceived threat of freedom: food options	9.23	2.88	9.14	2.86	9.38	2.87	9.14	2.94
Perceived threat of freedom: cafeteria board	8.31	3.00	7.86	2.94	8.69	3.06	8.27	2.98
Anger: food options	7.70	3.37	7.54	3.53	8.20	3.50	7.30	3.07
Anger: cafeteria board	7.35	3.36	6.98	3.46	7.75	3.56	7.22	3.06
Inertia	13.76	5.21	13.83	5.37	14.29	5.05	13.13	5.25

Means and standard deviations are displayed for the overall variable of time and per timepoint. T0 = baseline measure, T1 = post-measure, T2 = follow-up measure.

*Reactance.* A MANOVA was conducted to investigate the association between the intervention implementation over time and reactance (i.e., composed of the two anger subscales and the two perceived threat of freedom subscales). The multivariate tests revealed no significant overall effect for Time, Pillai’s Trace = 0.03, *F*(2, 444) = 0.86, *p* = 0.554, $\eta_{p}^{2}$ = 02. This suggests that reactance, as indicated by the four subscales, did not change at the post-measure and at the follow-up measure as compared to the baseline measure (see Table 1).

*Inertia.* Furthermore, to examine the association between the default intervention and inertia over time, an ANOVA was employed. The results revealed no significant main effect of time, *F*(2, 224) = 1.04, *p* = 0.357, $\eta_{p}^{2}$ = 0.01. This indicates that inertia did not increase nor decrease at the post-measure and the follow-up as compared to the baseline measure (see Table 1).

## Discussion

The current study investigated whether the introduction of a vegetarian default is associated with a long-term promotion in the selection of vegetarian items in a field experiment. Results indicate a strong significant interaction between time and the total number of food items sold, which was in line with our hypotheses. At baseline, no difference was found between the number of vegetarian and meat items sold. Yet, both right after implementation of the default as well as 10 weeks afterwards, the number of vegetarian items increased more than twofold, while the sales of meat items decreased by more than a third.

These findings are in line with, and expand upon, previous research (Campbell-Arvai et al., 2014; De Vaan et al., 2019; Gravert and Kurz, 2021; Hielkema et al., 2022; Meier et al., 2022; Taufik et al., 2022). In line with prior work, our results show that vegetarian defaults increase the demand for vegetarian items, whilst the demand for meat items decreases. Additionally, we expand upon prior research in two significant ways: First, this is the first study investigating the effects of a vegetarian default over a longer period (i.e., 16 weeks), which is notably longer than previous studies (e.g., Gravert and Kurz, 2021, and Taufik et al., 2022). This is important because it means that the default effect remains in place even after people realize meat options are still available. Second, our study contributes to the existing literature on vegetarian defaults and decision-making as prior research mainly concerned artificial choice scenarios, often in online or lab settings (e.g., Betz et al., 2022; Campbell-Arvai et al., 2014; De Vaan et al., 2019; Hielkema et al., 2022; Gravert and Kurz, 2021; Taufik et al., 2022). In contrast, our study examined real-life behaviour in a real-life setting. Therefore, our results can be generalized to real-life settings such as companies or institutions with a cafeteria more easily, and support people who are interested in promoting ecologically sustainable food choices in their work environment. This high external validity and societal relevance is perhaps best demonstrated by the fact that the university cafeteria which was the object of this study, transitioned into a fully vegetarian food assortment after learning about the results of the current study.

In addition to the behavioural data, we also collected survey data regarding reactance and inertia to explore potential psychological side-effects of a vegetarian default intervention. A more exploratory approach was adopted in our analysis of these data, as we were less sure about clear hypotheses and directions of effects. No evidence was found for any effect of the intervention on participants’ feelings of reactance and inertia, neither immediately after the intervention implementation, nor 10 weeks afterwards. Thus, the implementation of the vegetarian defaults could not be associated with significant changes in either reactance or inertia.

Regarding reactance, these findings align with earlier default interventions that found that vegetarian defaults are not related to a significant increase in reactance (Hertwig and Grüne-Yanoff, 2017; De Vaan et al., 2019). Another part of the literature stipulates that, along the lines of the change model (Lewin, 1947), reactance might increase shortly after intervention implementation and then decline again at the follow-up measurement due to acceptance of the new situation. Our results do not support this notion. A possible explanation for this might be that the individuals participating in the current study hold positive attitudes towards a vegetarian diet, and thus towards implementation of a vegetarian default, and showed therefore less reactance. However, this seems less likely given that reactance towards the food items was at a medium level across all three measurements. If positive attitudes were the main factor, reactance should have declined following intervention implementation. Thus, our research provides valuable reinforcement of existing knowledge indicating that freedom of choice is preserved when implementing vegetarian defaults and that vegetarian defaults are thus far not associated with a significant increase in reactance. It goes beyond current literature in such a way that these positive effects of the relation between vegetarian defaults and reactance (De Vaan et al., 2019) are established longitudinally in a real-world setting. This, in turn, improves the development of health, animal welfare, and sustainability improving strategies which do not evoke reactance.

Regarding inertia, our findings align with the notion that defaults do not increase nor decrease inertia across the three measures, which is in line with prior research (Joyner-Grantham et al., 2009; Knowles and Linn, 2004; Suri et al., 2013). Coupled with our observations in sales, we thus conclude that vegetarian defaults appear to be an effective strategy to encourage a shift towards a vegetarian diet among individuals who initially show inertia to such dietary changes.

## Limitations and future research

The findings of the current study, encompassing behavioural and survey data, collectively indicate that vegetarian defaults seem an effective strategy to reduce meat consumption in a cafeteria setting. Yet, we cannot state with absolute certainty that this can be achieved without provoking increased reactance since the survey responses were not necessarily provided by the customers that bought items and thus contributed to the behavioural data. We controlled for this by including regular patronage of the cafeteria as a criterion for participation. Hence, future studies could attempt to achieve tighter control between behavioural and self-report measures either by conducting research in a canteen where it would not cause disruption, or by implementing a more stringent inclusion criterion, such as requiring having purchased a meal that day, instead of merely being a regular customer. Nevertheless, as results of the study of De Vaan et al. (2019) did have the direct link between reactance data and item choice data, and did not show an increase in reactance, it seems less likely that reactance would have increased after exposure to the vegetarian default intervention.

Another limitation of our study, and inherent to field studies in general, is the limited control over the available food items. The availability and variety of both vegetarian and meat items changed over time when the intervention was implemented; specifically, the number of vegetarian items and its variety increased whilst those of meat items decreased. This adjustment was logical, as the canteen management aimed to avoid food waste and financial loss. Since the number of vegetarian items and its variety went up, this alone might have contributed to an increase in sales of these items, independent of the default set-up (Sethuraman et al., 2022). Ideally, future studies should maintain the number and variety of items constant across all time points, isolating the intervention’s impact. If feasible, future studies should investigate this set-up, particularly if they can cover the financial costs for the participating canteen. However, the field study by Taufik et al. (2022) demonstrated that maintaining all options constant, except for one changed to a vegetarian default, significantly boosted its selection. This provides compelling evidence that the observed sales increase in our study might be attributed more to the impact of the vegetarian default set-up than to the typical effect of increased assortment.

Additionally, it is important to note that we chose not to exclude data from vegan and vegetarian participants for several reasons. First, including these diets provided a more complete representation of the general population, acknowledging the variability in dietary preferences across different settings. Even though the prevalence of vegetarians in our study was higher than in the general population, we believe that in various real-world settings, such prevalence fluctuates depending on the context. In addition, it is crucial to explore settings beyond the specific, highly educated population our study focused on, as this ensures results are applicable across a broader demographic spectrum, encompassing varied age groups, professions, and cultural backgrounds. Therefore, future research should explore this variability in diverse societal settings and with different target groups. From a health and environmental perspective, it is especially interesting to see whether people with a higher meat consumption respond similarly to such an intervention. Second, excluding vegetarian of vegan diets would not necessarily mean that the results differed. Our decision was informed by the nature of our survey, which focused on reactance related to the canteen’s board and visible food items rather than specific food choices. This approach allowed us to capture potential reactance among vegetarians and vegans, who, despite their dietary preferences, might still experience reactance against perceived paternalism. However, while vegetarians and vegans inherently exhibit the desired behaviour of not consuming meat and thus are less likely to experience inertia, our study’s design, particularly the limitation regarding the missing connection between the survey and behavioural data, necessitated including all diets for a comprehensive analysis. Related to this, it would have been valuable to specifically examine the out-of-home buying and eating behaviour of different groups during the observation period. Thus, future studies should consider the possible effects of various diets and aim to represent a broader spectrum of society to improve generalisability.

Finally, since the effects of vegetarian defaults are now established, applying similar strategies with plant-based defaults might be a next step in reducing the carbon footprint of cafeterias (Fehér et al., 2020; Willett et al., 2019). However, it is important to proceed with caution as our findings cannot just be generalized to vegan default nudges. More specifically, while our research suggests that vegetarian defaults do not increase resistance or decrease sales, utilising plant-based defaults might lead to different results (De Groeve and Rosenfeld, 2022; Gregson et al., 2022; MacInnis and Hodson, 2017). Additionally, we do not know whether at-home eating was impacted by the food choices of customers, and it could be that people will eat more meat/fish (a rebound effect) or less meat/fish (a generalization effect). Recent research suggests that generalization effects (so-called positive spill-over effects) could be found in relation to more sustainable meat consumption in different settings (e.g., Graves and Roelich, 2021; Lanzini and Thøgersen, 2014; Nilsson et al., 2016). Therefore, future studies need to investigate whether adopting a plant-based default could potentially lead to similar positive results as those found in our study, or if it could instead lead to increased reactance or negatively impact sales. This would offer crucial insights for implementing such initiatives effectively.

## Conclusion

This is the first field study that captures real behaviour in a natural setting, utilizes a large dataset, and provides longitudinal insights. In addition, this is the first study to explore the potential side-effects of a default nudging intervention by examining the relation with the psychological constructs of reactance and inertia. Our research helps to understand how nudging in the form of vegetarian defaults can effectively be utilised. The results are crucial for understanding the nuances of reactance and inertia in the context of food changes and organisational policies, offering a valuable foundation for future research and practical applications in similar settings. Based on our work, concerns about declining sales seem unwarrant. Moreover, our research aligns with the growing public and corporate interest in ecological sustainability, animal welfare, and health. We recommend to further investigate how vegetarian and vegan defaults can be established in canteens on a larger scale to support efforts to offer sustainable food choices in the public sphere. Our study suggests that implementing vegetarian defaults might be a subtle, non-disruptive, and cost-efficient, yet effective and feasible behaviour change strategy. Such a policy would not require drastic changes from costumers and might have the potential to support a positive impact on ecologically sustainable and health-conscious choices.

## Data Availability

The raw data supporting the conclusions of this article will be made available by the authors, without undue reservation.

## Footnotes

^1^https://osf.io/6fsbj/.

^2^The survey also contained several items regarding the cafeteria’s pricing, variety, reasons for purchasing food items, as well as respondents’ own dietary restrictions. These data were solely collected for collaboration with the cafeteria and, consequently, were not analysed further.
